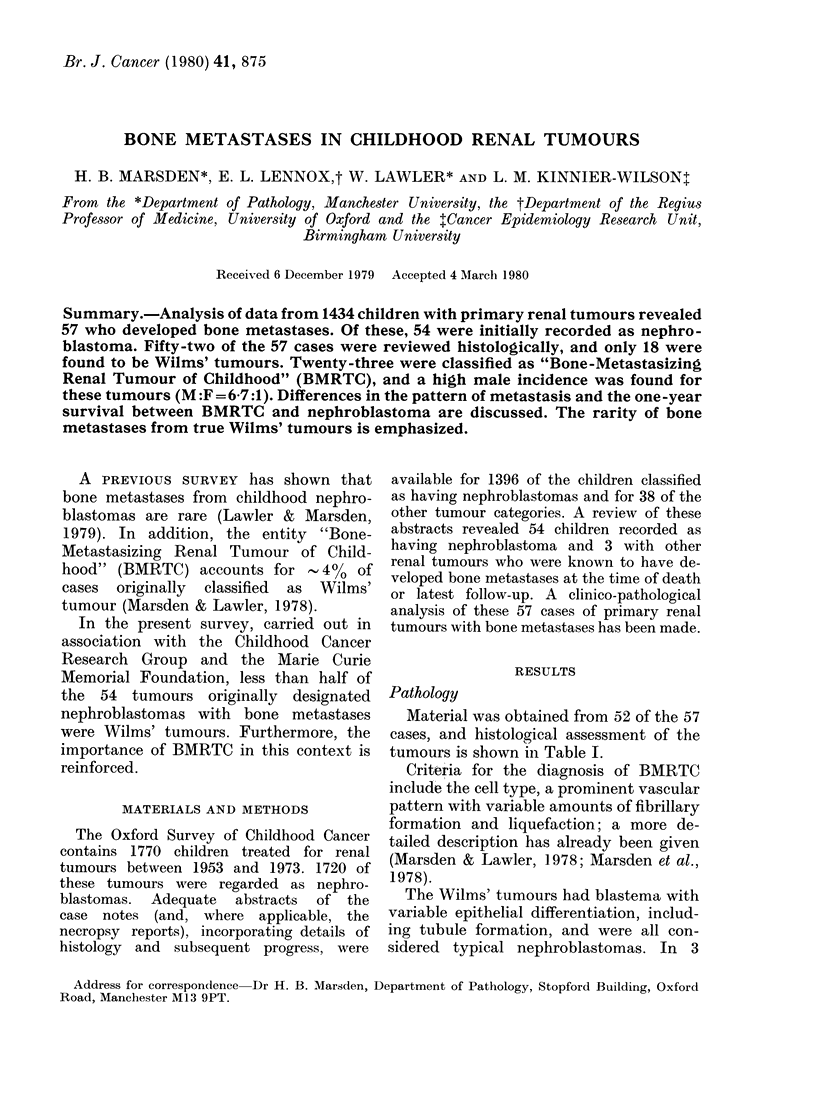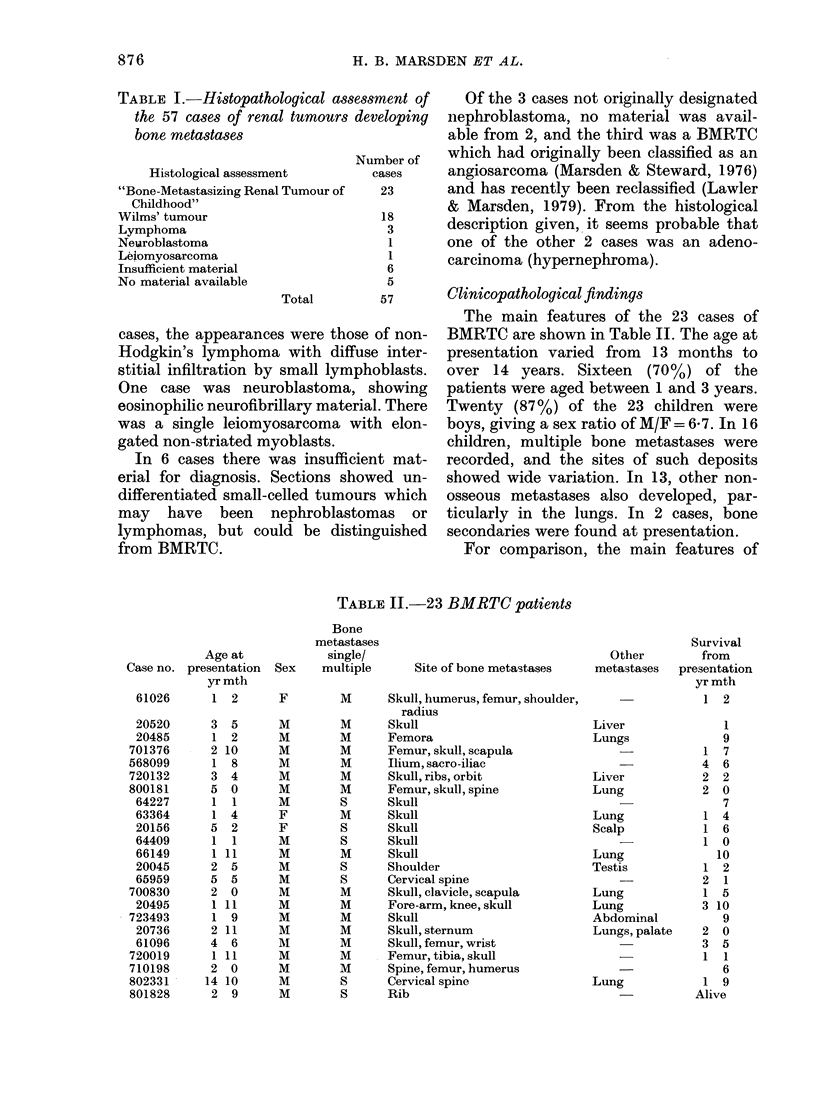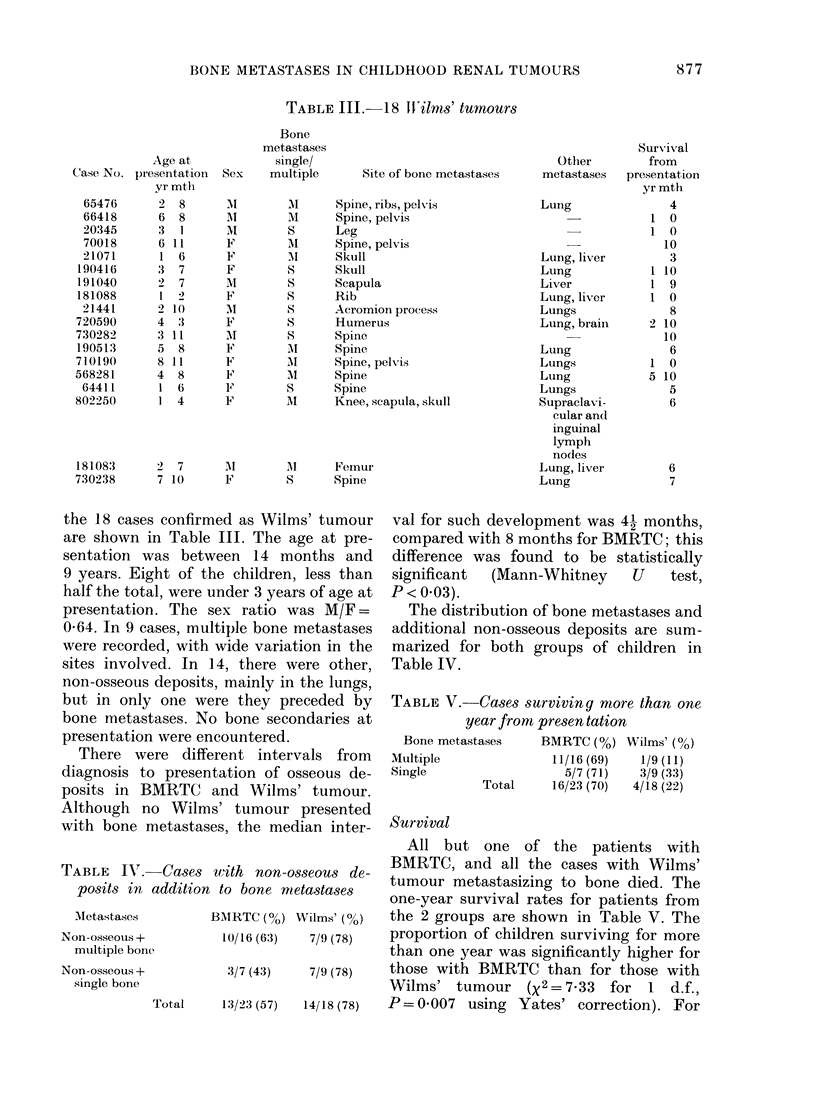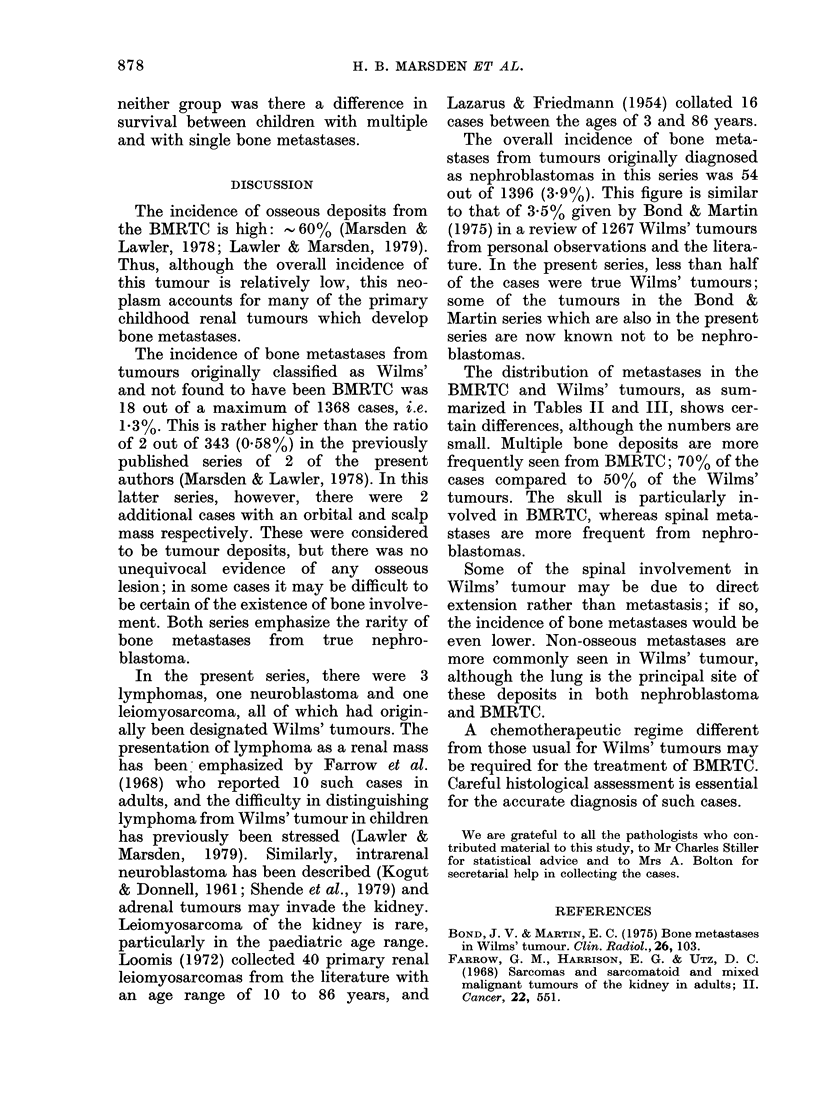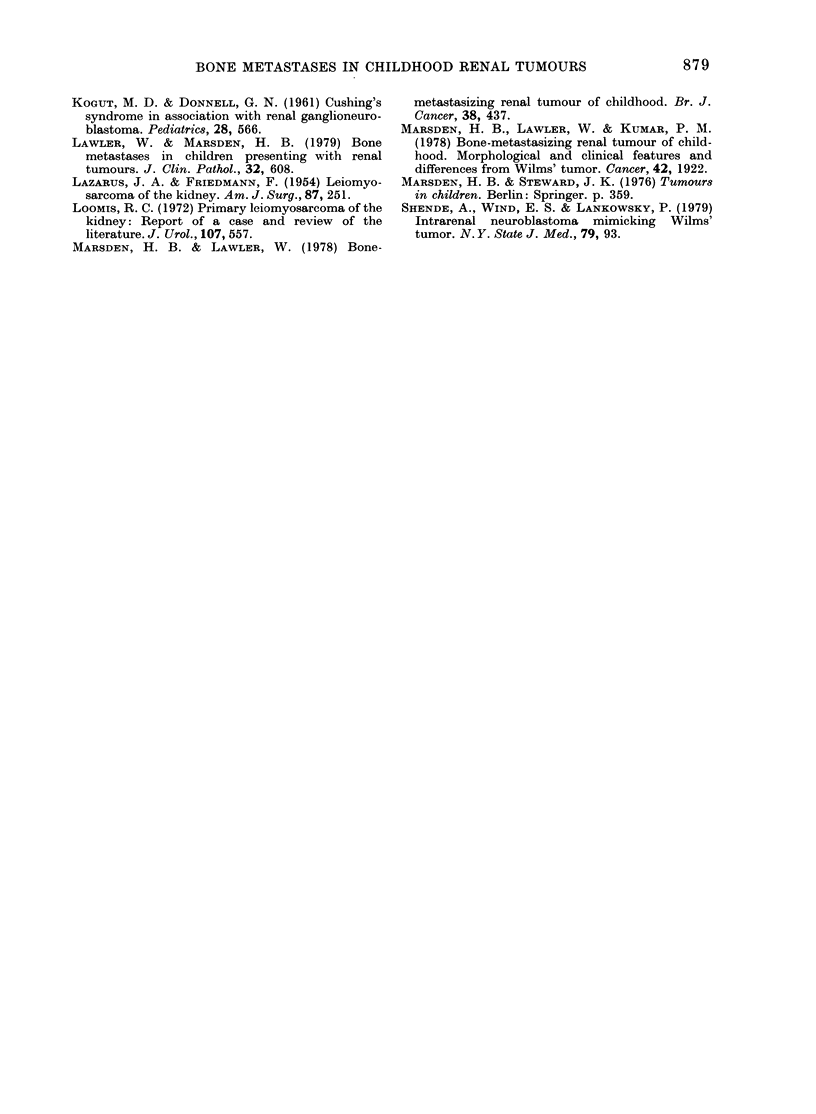# Bone metastases in childhood renal tumours.

**DOI:** 10.1038/bjc.1980.163

**Published:** 1980-06

**Authors:** H. B. Marsden, E. L. Lennox, W. Lawler, L. M. Kinnier-Wilson

## Abstract

Analysis of data from 1434 children with primary renal tumours revealed 57 who developed bone metastases. Of these, 54 were initially recorded as nephroblastoma. Fifty-two of the 57 cases were reviewed histologically, and only 18 were found to be Wilms' tumours. Twenty-three were classified as "Bone-Metastasizing Renal Tumour of Childhood" (BMRTC), and a high male incidence was found for these tumours (M:F=6.7:1). Differences in the pattern of metastasis and the one-year survival between BMRTC and nephroblastoma are discussed. The rarity of bone metastases from true Wilms' tumours is emphasized.


*Br. J. Cancer* (1980) **41**, 875

### BONE METASTASES IN CHILDHOOD RENAL TUMOURS

H. B. MARSDEN*, E. L. LENNOX,† W. LAWLER* AND L. M. KINNIER-WILSON‡

*From the *Department of Pathology, Manchester University, the †Department of the Regius Professor of Medicine, University of Oxford and the ‡Cancer Epidemiology Research Unit, Birmingham University*

Received 6 December 1979 Accepted 4 March 1980

**Summary.—Analysis of data from 1434 children with primary renal tumours revealed 57 who developed bone metastases. Of these, 54 were initially recorded as nephroblastoma. Fifty-two of the 57 cases were reviewed histologically, and only 18 were found to be Wilms' tumours. Twenty-three were classified as "Bone-Metastasizing Renal Tumour of Childhood" (BMRTC), and a high male incidence was found for these tumours (M:F = 6·7:1). Differences in the pattern of metastasis and the one-year survival between BMRTC and nephroblastoma are discussed. The rarity of bone metastases from true Wilms' tumours is emphasized.**

A PREVIOUS SURVEY has shown that bone metastases from childhood nephroblastomas are rare (Lawler & Marsden, 1979). In addition, the entity "Bone-Metastasizing Renal Tumour of Childhood" (BMRTC) accounts for ~4% of cases originally classified as Wilms' tumour (Marsden & Lawler, 1978).

In the present survey, carried out in association with the Childhood Cancer Research Group and the Marie Curie Memorial Foundation, less than half of the 54 tumours originally designated nephroblastomas with bone metastases were Wilms' tumours. Furthermore, the importance of BMRTC in this context is reinforced.

### MATERIALS AND METHODS

The Oxford Survey of Childhood Cancer contains 1770 children treated for renal tumours between 1953 and 1973. 1720 of these tumours were regarded as nephroblastomas. Adequate abstracts of the case notes (and, where applicable, the necropsy reports), incorporating details of histology and subsequent progress, were available for 1396 of the children classified as having nephroblastomas and for 38 of the other tumour categories. A review of these abstracts revealed 54 children recorded as having nephroblastoma and 3 with other renal tumours who were known to have developed bone metastases at the time of death or latest follow-up. A clinico-pathological analysis of these 57 cases of primary renal tumours with bone metastases has been made.

### RESULTS

### *Pathology*

Material was obtained from 52 of the 57 cases, and histological assessment of the tumours is shown in Table I.

Criteria for the diagnosis of BMRTC include the cell type, a prominent vascular pattern with variable amounts of fibrillary formation and liquefaction; a more detailed description has already been given (Marsden & Lawler, 1978; Marsden *et al.*, 1978).

The Wilms' tumours had blastema with variable epithelial differentiation, including tubule formation, and were all considered typical nephroblastomas. In 3

Address for correspondence—Dr H. B. Marsden, Department of Pathology, Stopford Building, Oxford Road, Manchester M13 9PT.

TABLE I.—*Histopathological assessment of the 57 cases of renal tumours developing bone metastases*

| Histological assessment | Number of cases |
|---|---|
| "Bone-Metastasizing Renal Tumour of Childhood" | 23 |
| Wilms' tumour | 18 |
| Lymphoma | 3 |
| Neuroblastoma | 1 |
| Leiomyosarcoma | 1 |
| Insufficient material | 6 |
| No material available | 5 |
| Total | 57 |

cases, the appearances were those of non-Hodgkin's lymphoma with diffuse interstitial infiltration by small lymphoblasts. One case was neuroblastoma, showing eosinophilic neurofibrillary material. There was a single leiomyosarcoma with elongated non-striated myoblasts.

In 6 cases there was insufficient material for diagnosis. Sections showed undifferentiated small-celled tumours which may have been nephroblastomas or lymphomas, but could be distinguished from BMRTC.

Of the 3 cases not originally designated nephroblastoma, no material was available from 2, and the third was a BMRTC which had originally been classified as an angiosarcoma (Marsden & Steward, 1976) and has recently been reclassified (Lawler & Marsden, 1979). From the histological description given, it seems probable that one of the other 2 cases was an adenocarcinoma (hypernephroma).

*Clinicopathological findings*

The main features of the 23 cases of BMRTC are shown in Table II. The age at presentation varied from 13 months to over 14 years. Sixteen (70%) of the patients were aged between 1 and 3 years. Twenty (87%) of the 23 children were boys, giving a sex ratio of M/F = 6·7. In 16 children, multiple bone metastases were recorded, and the sites of such deposits showed wide variation. In 13, other non-osseous metastases also developed, particularly in the lungs. In 2 cases, bone secondaries were found at presentation.

For comparison, the main features of

TABLE II.—23 *BMRTC patients*

| Case no. | Age at presentation yr mth | Sex | Bone metastases single/ multiple | Site of bone metastases | Other metastases | Survival from presentation yr mth |
|---|---|---|---|---|---|---|
| 61026 | 1 2 | F | M | Skull, humerus, femur, shoulder, radius | — | 1 2 |
| 20520 | 3 5 | M | M | Skull | Liver | 1 |
| 20485 | 1 2 | M | M | Femora | Lungs | 9 |
| 701376 | 2 10 | M | M | Femur, skull, scapula | — | 1 7 |
| 568099 | 1 8 | M | M | Ilium, sacro-iliac | — | 4 6 |
| 720132 | 3 4 | M | M | Skull, ribs, orbit | Liver | 2 2 |
| 800181 | 5 0 | M | M | Femur, skull, spine | Lung | 2 0 |
| 64227 | 1 1 | M | S | Skull | — | 7 |
| 63364 | 1 4 | F | M | Skull | Lung | 1 4 |
| 20156 | 5 2 | F | S | Skull | Scalp | 1 6 |
| 64409 | 1 1 | M | S | Skull | — | 1 0 |
| 66149 | 1 11 | M | M | Skull | Lung | 10 |
| 20045 | 2 5 | M | S | Shoulder | Testis | 1 2 |
| 65959 | 5 5 | M | S | Cervical spine | — | 2 1 |
| 700830 | 2 0 | M | M | Skull, clavicle, scapula | Lung | 1 5 |
| 20495 | 1 11 | M | M | Fore-arm, knee, skull | Lung | 3 10 |
| 723493 | 1 9 | M | M | Skull | Abdominal | 9 |
| 20736 | 2 11 | M | M | Skull, sternum | Lungs, palate | 2 0 |
| 61096 | 4 6 | M | M | Skull, femur, wrist | — | 3 5 |
| 720019 | 1 11 | M | M | Femur, tibia, skull | — | 1 1 |
| 710198 | 2 0 | M | M | Spine, femur, humerus | — | 6 |
| 802331 | 14 10 | M | S | Cervical spine | Lung | 1 9 |
| 801828 | 2 9 | M | S | Rib | — | Alive |

TABLE III.—18 *Wilms' tumours*

| Case No. | Age at presentation yr mth | Sex | Bone metastases single/ multiple | Site of bone metastases | Other metastases | Survival from presentation yr mth |
|---|---|---|---|---|---|---|
| 65476 | 2 8 | M | M | Spine, ribs, pelvis | Lung | 4 |
| 66418 | 6 8 | M | M | Spine, pelvis | — | 1 0 |
| 20345 | 3 1 | M | S | Leg | — | 1 0 |
| 70018 | 6 11 | F | M | Spine, pelvis | — | 10 |
| 21071 | 1 6 | F | M | Skull | Lung, liver | 3 |
| 190416 | 3 7 | F | S | Skull | Lung | 1 10 |
| 191040 | 2 7 | M | S | Scapula | Liver | 1 9 |
| 181088 | 1 2 | F | S | Rib | Lung, liver | 1 0 |
| 21441 | 2 10 | M | S | Acromion process | Lungs | 8 |
| 720590 | 4 3 | F | S | Humerus | Lung, brain | 2 10 |
| 730282 | 3 11 | M | S | Spine | — | 10 |
| 190513 | 5 8 | F | M | Spine | Lung | 6 |
| 710190 | 8 11 | F | M | Spine, pelvis | Lungs | 1 0 |
| 568281 | 4 8 | F | M | Spine | Lung | 5 10 |
| 64411 | 1 6 | F | S | Spine | Lungs | 5 |
| 802250 | 1 4 | F | M | Knee, scapula, skull | Supraclavicular and inguinal lymph nodes | 6 |
| 181083 | 2 7 | M | M | Femur | Lung, liver | 6 |
| 730238 | 7 10 | F | S | Spine | Lung | 7 |

the 18 cases confirmed as Wilms' tumour are shown in Table III. The age at presentation was between 14 months and 9 years. Eight of the children, less than half the total, were under 3 years of age at presentation. The sex ratio was M/F = 0·64. In 9 cases, multiple bone metastases were recorded, with wide variation in the sites involved. In 14, there were other, non-osseous deposits, mainly in the lungs, but in only one were they preceded by bone metastases. No bone secondaries at presentation were encountered.

There were different intervals from diagnosis to presentation of osseous deposits in BMRTC and Wilms' tumour. Although no Wilms' tumour presented with bone metastases, the median interval for such development was $4\frac{1}{2}$ months, compared with 8 months for BMRTC; this difference was found to be statistically significant (Mann-Whitney *U* test, $P < 0{\cdot}03$).

The distribution of bone metastases and additional non-osseous deposits are summarized for both groups of children in Table IV.

TABLE IV.—*Cases with non-osseous deposits in addition to bone metastases*

| Metastases | BMRTC (%) | Wilms' (%) |
|---|---|---|
| Non-osseous + multiple bone | 10/16 (63) | 7/9 (78) |
| Non-osseous + single bone | 3/7 (43) | 7/9 (78) |
| Total | 13/23 (57) | 14/18 (78) |

TABLE V.—*Cases surviving more than one year from presentation*

| Bone metastases | BMRTC (%) | Wilms' (%) |
|---|---|---|
| Multiple | 11/16 (69) | 1/9 (11) |
| Single | 5/7 (71) | 3/9 (33) |
| Total | 16/23 (70) | 4/18 (22) |

*Survival*

All but one of the patients with BMRTC, and all the cases with Wilms' tumour metastasizing to bone died. The one-year survival rates for patients from the 2 groups are shown in Table V. The proportion of children surviving for more than one year was significantly higher for those with BMRTC than for those with Wilms' tumour ($\chi^2 = 7{\cdot}33$ for 1 d.f., $P = 0{\cdot}007$ using Yates' correction). For neither group was there a difference in survival between children with multiple and with single bone metastases.

### DISCUSSION

The incidence of osseous deposits from the BMRTC is high: ~60% (Marsden & Lawler, 1978; Lawler & Marsden, 1979). Thus, although the overall incidence of this tumour is relatively low, this neoplasm accounts for many of the primary childhood renal tumours which develop bone metastases.

The incidence of bone metastases from tumours originally classified as Wilms' and not found to have been BMRTC was 18 out of a maximum of 1368 cases, *i.e.* 1·3%. This is rather higher than the ratio of 2 out of 343 (0·58%) in the previously published series of 2 of the present authors (Marsden & Lawler, 1978). In this latter series, however, there were 2 additional cases with an orbital and scalp mass respectively. These were considered to be tumour deposits, but there was no unequivocal evidence of any osseous lesion; in some cases it may be difficult to be certain of the existence of bone involvement. Both series emphasize the rarity of bone metastases from true nephroblastoma.

In the present series, there were 3 lymphomas, one neuroblastoma and one leiomyosarcoma, all of which had originally been designated Wilms' tumours. The presentation of lymphoma as a renal mass has been emphasized by Farrow *et al.* (1968) who reported 10 such cases in adults, and the difficulty in distinguishing lymphoma from Wilms' tumour in children has previously been stressed (Lawler & Marsden, 1979). Similarly, intrarenal neuroblastoma has been described (Kogut & Donnell, 1961; Shende *et al.*, 1979) and adrenal tumours may invade the kidney. Leiomyosarcoma of the kidney is rare, particularly in the paediatric age range. Loomis (1972) collected 40 primary renal leiomyosarcomas from the literature with an age range of 10 to 86 years, and Lazarus & Friedmann (1954) collated 16 cases between the ages of 3 and 86 years.

The overall incidence of bone metastases from tumours originally diagnosed as nephroblastomas in this series was 54 out of 1396 (3·9%). This figure is similar to that of 3·5% given by Bond & Martin (1975) in a review of 1267 Wilms' tumours from personal observations and the literature. In the present series, less than half of the cases were true Wilms' tumours; some of the tumours in the Bond & Martin series which are also in the present series are now known not to be nephroblastomas.

The distribution of metastases in the BMRTC and Wilms' tumours, as summarized in Tables II and III, shows certain differences, although the numbers are small. Multiple bone deposits are more frequently seen from BMRTC; 70% of the cases compared to 50% of the Wilms' tumours. The skull is particularly involved in BMRTC, whereas spinal metastases are more frequent from nephroblastomas.

Some of the spinal involvement in Wilms' tumour may be due to direct extension rather than metastasis; if so, the incidence of bone metastases would be even lower. Non-osseous metastases are more commonly seen in Wilms' tumour, although the lung is the principal site of these deposits in both nephroblastoma and BMRTC.

A chemotherapeutic regime different from those usual for Wilms' tumours may be required for the treatment of BMRTC. Careful histological assessment is essential for the accurate diagnosis of such cases.

We are grateful to all the pathologists who contributed material to this study, to Mr Charles Stiller for statistical advice and to Mrs A. Bolton for secretarial help in collecting the cases.

### REFERENCES

BOND, J. V. & MARTIN, E. C. (1975) Bone metastases in Wilms' tumour. *Clin. Radiol.*, **26**, 103.

FARROW, G. M., HARRISON, E. G. & UTZ, D. C. (1968) Sarcomas and sarcomatoid and mixed malignant tumours of the kidney in adults; II. *Cancer*, **22**, 551.

KOGUT, M. D. & DONNELL, G. N. (1961) Cushing's syndrome in association with renal ganglioneuroblastoma. *Pediatrics*, **28**, 566.

LAWLER, W. & MARSDEN, H. B. (1979) Bone metastases in children presenting with renal tumours. *J. Clin. Pathol.*, **32**, 608.

LAZARUS, J. A. & FRIEDMANN, F. (1954) Leiomyosarcoma of the kidney. *Am. J. Surg.*, **87**, 251.

LOOMIS, R. C. (1972) Primary leiomyosarcoma of the kidney: Report of a case and review of the literature. *J. Urol.*, **107**, 557.

MARSDEN, H. B. & LAWLER, W. (1978) Bone-metastasizing renal tumour of childhood. *Br. J. Cancer*, **38**, 437.

MARSDEN, H. B., LAWLER, W. & KUMAR, P. M. (1978) Bone-metastasizing renal tumour of childhood. Morphological and clinical features and differences from Wilms' tumor. *Cancer*, **42**, 1922.

MARSDEN, H. B. & STEWARD, J. K. (1976) *Tumours in children*. Berlin: Springer. p. 359.

SHENDE, A., WIND, E. S. & LANKOWSKY, P. (1979) Intrarenal neuroblastoma mimicking Wilms' tumor. *N.Y. State J. Med.*, **79**, 93.